# Investigation on Risk Factors of Ventilator-Associated Pneumonia in Acute Cerebral Hemorrhage Patients in Intensive Care Unit

**DOI:** 10.1155/2017/7272080

**Published:** 2017-12-17

**Authors:** Li Chang, Yun Dong, Ping Zhou

**Affiliations:** Department of Emergency Intensive Care Unit, Sichuan Province People's Hospital, Chengdu, Sichuan, China

## Abstract

Ventilator-associated pneumonia (VAP) is a predominant factor of pulmonary infection. We analyzed the risk factors of VAP with acute cerebral hemorrhage in intensive care unit (ICU) by univariate and multivariate logistic regression analyses. After comparison of 197 cases of the VAP and non-VAP patients, we found that age > 65 years (*P* = 0.003), smoke (*P* = 0.003), coronary heart disease (*P* = 0.005), diabetes (*P* = 0.001), chronic obstructive pulmonary disease (COPD) (*P* = 0.002), ICU and hospital stay (*P* = 0.01), and days on mechanical ventilation (*P* = 0.01) were significantly different, indicating that they are risk factors of VAP. All the age > 65 years (OR = 3.350, 95% CI = 1.936–5.796, *P* ≤ 0.001), smoke (OR = 3.206, 95% CI = 1.909–5.385, *P* ≤ 0.001), coronary heart disease (OR = 3.179, 95% CI = 1.015–4.130, *P* = 0.017), diabetes (OR = 5.042, 95% CI = 3.518–7.342, *P* ≤ 0.001), COPD (OR = 1.942, 95% CI = 1.258–2.843, *P* = 0.012), ICU and hospital stay (OR = 2.34, 95% CI = 1.145–3.892, *P* = 0.038), and days on mechanical ventilation (OR = 1.992, 95% CI = 1.107–3.287, *P* = 0.007) are independent risk factors of VAP. After observation of patients with 6 months of follow-up, the BI score was significantly lower in VAP than that in non-VAP, and the rebleeding rate and mortality rate were significantly higher in VAP than those in non-VAP. Thus, the prognosis of the patients with acute cerebral hemorrhage and VAP in ICU is poor.

## 1. Introduction

Mechanical ventilation is a supportive treatment for the maintenance of respiratory function [1, 2], which was used in the perioperative period and all types of critically ill patients in the rescue phase, especially in the occurrence of acute lung injury, acute respiratory distress syndrome, and acute cerebral hemorrhage [3–5]. Mechanical ventilation simulates physiological respiration, which ensures oxygen supply and meets the needs of oxygen [4]. However, mechanical ventilation is different from the physiological ventilation model, which can act as a factor causing mechanical ventilation-related lung injury [6, 7]. Clinically, during the use of mechanical ventilation, even if the patient's previous lung function is normal, there may be local inflammatory response, a certain degree of lung injury, and even acute lung injury [8–10].

Pulmonary infection is one of the most common complications in patients with acute cerebral hemorrhage, which directly affects the prognosis of those patients [11–13]. Ventilator-associated pneumonia (VAP) is defined as the onset of pneumonia in patients with tracheal intubation or tracheotomy after 48 h of mechanical ventilation [14, 15]. After withdrawal or extubation, pneumonia within 48 h is still VAP. VAP is the most serious type of hospital-acquired pneumonia, with the incidence rate of 15% to 60% and the mortality rate of 25% to 76%, and is also the major risk factor of patients in intensive care unit (ICU) [16–18]. VAP is a predominant factor of pulmonary infection in patients with acute cerebral hemorrhage. VAP not only prolongs the hospital stay but also increases the cost of treatment. Acute cerebral hemorrhage can directly affect the prognosis of patients, prolong the hospital stay, increase the medical costs, and accelerate the deterioration of the disease, so it is of great significance to prevent VAP occurrence by early identification of the risk factors of acute cerebral hemorrhagic-related pneumonia and control the predictable factors in the safe range [17, 19, 20].

In this study, the clinical data of patients with acute cerebral hemorrhage in our hospital emergency intensive care unit in recent years were retrospectively analyzed to find out the risk factors of VAP in acute cerebral hemorrhage patients. Our study provides the theoretical basis for early clinical decision and might improve the life expectancy and quality of life of patients with acute cerebral hemorrhage.

## 2. Materials and Methods

### 2.1. Clinical Data

One hundred ninety-seven cases of patients with acute cerebral hemorrhage with or without mechanical ventilation from December 2014 to December 2016 in the Emergency Intensive Care Unit, Sichuan Provincial People's Hospital, China, were enrolled after approval by the Medical Ethic Committee of Sichuan Provincial People's Hospital. Written informed consents conforming to the tenets of the Declaration of Helsinki were obtained from each participant prior to the study. The acute cerebral hemorrhage of patients was confirmed by skull CT and head MRI examination. All the patients are in line with the guideline of diagnosis and treatment of hospital-acquired pneumonia in China made by the Chinese Society of Respiratory Diseases.

### 2.2. Data Analysis

A retrospective analysis of the clinical data of 197 patients with acute cerebral hemorrhage was performed, including age, gender, past history (smoke, coronary heart disease, hypertension, and diabetes), COPD, ICU and hospital stay, and days on mechanical ventilation. Moreover, the correlation analysis of the indicators with VAP was performed.

### 2.3. Statistical Analysis

Using SPSS19.0 software for statistical analysis, count data were compared by *χ*^2^ test and Fisher's exact probability test for a table of % frequency data, and the influential factors of VAP were analyzed by univariate and multivariate logistic regression analyses. *P* < 0.05 was statistically significant.

## 3. Results

### 3.1. Univariate Logistic Regression Analysis of the Risk Factors of VAP

In the patients with ventilator-associated pneumonia (VAP), there were 46 males and 36 females, aged from 26 to 79, with an average age of 55.47 ± 13.53 years (Table 1). In the non-VAP group, there were 66 males and 49 females, aged from 27 to 77, with an average age of 56.52 ± 16.23 years. After comparison of the VAP and non-VAP patients with acute cerebral hemorrhage, we found that age > 65 years (*P*=0.003), smoke (*P*=0.003), coronary heart disease (*P*=0.005), diabetes (*P*=0.001), chronic obstructive pulmonary disease (COPD) (*P*=0.002), ICU and hospital stay (*P*=0.01), and days on mechanical ventilation (*P*=0.01) were significantly different, indicating that they are risk factors of VAP.

### 3.2. Multivariate Logistic Regression Analysis of the Risk Factors of VAP

The multivariate logistic regression analysis revealed that all the age > 65 years (OR = 3.350, 95% CI = 1.936–5.796, *P* ≤ 0.001), smoke (OR = 3.206, 95% CI = 1.909–5.385, *P* ≤ 0.001), coronary heart disease (OR = 3.179, 95% CI = 1.015–4.130, *P*=0.017), diabetes (OR = 5.042, 95% CI = 3.518–7.342, *P* ≤ 0.001), COPD (OR = 1.942, 95% CI = 1.258–2.843, *P*=0.012), ICU and hospital stay (OR = 2.34, 95% CI = 1.145–3.892, *P*=0.038), and days on mechanical ventilation (OR = 1.992, 95% CI = 1.107–3.287, *P*=0.007) are independent risk factors of VAP (Table 2).

### 3.3. The Mortality Rate and Rebleeding Rate in Patients with VAP Were Higher than Those Without VAP

After observation of patients with 6 months of follow-up, the BI score, rebleeding rate, and mortality rate were calculated. The BI score was significantly lower in VAP than that in non-VAP (*P*=0.003). The rebleeding rate and mortality rate were significantly higher in VAP than those in non-VAP (*P*=0.002 for rebleeding rate and *P*=0.01 for mortality rate) (Table 3).

## 4. Discussion

With the accelerated process of aging, the incidence of cerebrovascular disease in China increased with year and has the trend of rejuvenation, which is a serious threat to people's health [21]. Ventilator-associated pneumonia (VAP) is one of the most common complications after treatment of acute cerebral hemorrhage [3–5]. The serious pulmonary infection directly affects the prognosis of patients and even leads to death. Cerebral hemorrhage is a common disease that seriously damages people's health and life. The cerebral hemorrhage patients with more serious illness often entered the ICU. The study showed that patients are prone to VAP. This study showed 197 cases of acute cerebral hemorrhage patients. Among them, 82 cases are VAP, with the incidence of 41.6%, showing a very high incidence of VAP in ICU patients with acute cerebral hemorrhage. This might be due to the occurrence of a stress state in patients with acute cerebral hemorrhage, which increased the susceptibility to infection of VAP by decreasing the body resistance, inducing intracranial hypertension, affecting the thalamic function and the autonomic nerve function disorder, and inducing pulmonary hypertension and pulmonary capillary congestion. In addition, ICU is the gathering place of critically ill patients and the most serious hospital infection [22, 23]. We observed that the BI score was significantly lower in VAP than that in non-VAP. The rebleeding rate and mortality rate were significantly higher in VAP than those in non-VAP. It was suggested that VAP can seriously affect the prognosis of patients with acute cerebral hemorrhage; therefore, early clinical prevention has an important clinical value in patients suffering from cerebral hemorrhage VAP.

Our results showed that age > 65 years, smoke, coronary heart disease, diabetes, chronic obstructive pulmonary disease (COPD), ICU and hospital stay, and days on mechanical ventilation were risk factors of VAP. The respiratory physiological function in the elderly decreased gradually with the increase of age, the muscle of the respiratory muscle gradually shrank, the elasticity of the lung tissue decreased gradually, and the cough and expectoration movement decreased significantly [24–26]. Meanwhile, the respiratory mucosal atrophy and mucinous gland functions of the patients with senile cerebral hemorrhage were decreased significantly, thus reducing the local defense function of the respiratory tract which easily leads to VAP infection. Smoking can lead to damage to the respiratory system, resulting in shorter respiratory tract cilia, irregular, and ciliary movement disorders, to reduce the local defense capacity of the respiratory tract, which also easily leads to the occurrence of VAP. ICU time and longer duration of mechanical ventilation are risk factors of acquired pneumonia in ICU patients suffering from acute cerebral hemorrhage. This is because severe infection in ICU to the patients occurred more easily in the hospital, and the use of a respirator is a risk factor of VAP [15, 27]. The number of days of hospitalization in the ICU and the increase in the number of days of ventilator use in patients with acute cerebral hemorrhage linearly increased the incidence of infection. It should be strictly controlled in ICU and during the use of mechanical ventilation to reduce the incidence of VAP.

In summary, this study suggests that patients with VAP can cause adverse prognosis including increased morbidity of acute cerebral hemorrhage and increased bleeding rate. Age > 65 years, smoke, coronary heart disease, diabetes, chronic obstructive pulmonary disease (COPD), ICU and hospital stay, and days on mechanical ventilation were independent risk factors of VAP. It should be strictly controlled in ICU and during the use of mechanical ventilation to reduce the incidence of VAP.

## Figures and Tables

**Table 1:** Single-factor analysis of risk factors of ICU patients with acute cerebral hemorrhage.

Factors	VAP (*n* = 82)	Non-VAP (*n* = 115)	*χ* ^2^	*P*
Age			8.47	0.003
≤65	32 (38.6%)	78 (68.2%)		
>65	50 (61.4%)	37 (31.8%)		
Gender			2.53	0.08
Male	46 (56.1%)	66 (57.6%)		
Female	36 (43.9%)	49 (42.4%)		
Past history				
Smoke	47 (57.9%)	22 (18.9%)	9.72	0.003
Coronary heart disease	58 (71.6%)	78 (67.9%)	7.94	0.005
Hypertension	35 (42.1%)	46 (40.2%)	2.13	0.72
Diabetes	23 (28.1%)	22 (18.9%)	20.93	0.001
COPD	9 (10.5%)	10 (8.3%)	14.43	0.002
ICU and hospital stay	13.46 ± 5.92	10.35 ± 4.13	6.46	0.01
Days on mechanical ventilation	4.97 ± 1.35	2.56 ± 1.13	5.92	0.01

**Table 2:** Logistic regression analysis of risk factors of ICU patients with acute cerebral hemorrhage.

Factors	*β*	SE	OR (95% CI)	*P*
Age > 65 years	1.209	0.280	3.350 (1.936–5.796)	≤0.001
Smoke	1.165	0.265	3.206 (1.909–5.385)	≤0.001
Coronary heart disease	1.023	0.325	3.179 (1.015–4.130)	0.017
Diabetes	1.610	0.181	5.042 (3.518–7.342)	≤0.001
COPD	1.518	0.021	1.942 (1.258–2.843)	0.012
ICU and hospital stay	1.049	0.501	2.34 (1.145–3.892)	0.038
Days on mechanical ventilation	1.462	0.630	1.992 (1.107–3.287)	0.007

**Table 3:** Outcome and prognosis of ICU patients with acute cerebral hemorrhage.

Factors	VAP (*n* = 82)	Non-VAP (*n* = 115)		*P*
BI	52.47 ± 15.29	71.29 ± 19.26	16.26	0.001
Rebleeding rate	27 (32.9%)	18 (15.6%)	8.71	0.002
Mortality rate	21 (25.6%)	12 (10.4%)	6.76	0.01

## References

[1] Weiss B., Kaplan L. J. (2017). Oxygen therapeutics and mechanical ventilation advances. *Critical Care Clinics*.

[2] De Jong A., Chanques G., Jaber S. (2017). Mechanical ventilation in obese ICU patients: from intubation to extubation. *Critical Care*.

[3] Nakase H., Motoyama Y., Yamada S. (2016). Cerebral hemorrhage. *Nihon Rinsho*.

[4] Kumar S., Badrinath H. R. (2006). Early recombinant factor VIIa therapy in acute intracerebral hemorrhage: promising approach. *Neurology India*.

[5] Freeman W. D. (2015). Management of intracranial pressure. *Continuum*.

[6] Snow T. M., Brandon D. H. (2007). A nurse’s guide to common mechanical ventilation techniques and modes used in infants: nursing implications. *Advances in Neonatal Care*.

[7] Burns K. E., Adhikari N. K., Keenan S. P., Meade M. O. (2010). Noninvasive positive pressure ventilation as a weaning strategy for intubated adults with respiratory failure. *Cochrane Database of Systematic Reviews*.

[8] Wang W. F., Liu S., Xu B. (2017). A study of the protective effect and mechanism of ketamine on acute lung injury induced by mechanical ventilation. *European Review for Medical and Pharmacological Sciences*.

[9] Namuduri A. V., Heras G., Mi J. (2017). A proteomic approach to identify alterations in the SUMO network during controlled mechanical ventilation in rat diaphragm muscle. *Molecular & Cellular Proteomics*.

[10] Fot E. V., Izotova N. N., Yudina A. S., Smetkin A. A., Kuzkov V. V., Kirov M. Y. (2017). Automated weaning from mechanical ventilation after off-pump coronary artery bypass grafting. *Frontiers in Medicine*.

[11] Plummer M. P., Young A. M. H., O’Leary R. (2017). Probable catastrophic antiphospholipid syndrome with intracerebral hemorrhage secondary to Epstein-Barr viral infection. *Neurocritical Care*.

[12] Feng Y., He J., Liu B., Yang L., Wang Y. (2016). Endoscope-assisted keyhole technique for hypertensive cerebral hemorrhage in elderly patients: a randomized controlled study in 184 patients. *Turkish Neurosurgery*.

[13] Cai G., Shang L., Liu K. (2015). Remodeling of cross electro-nape-acupuncture on cough reflex in patients with tracheotomy after cerebral hemorrhage: a randomized controlled trial. *Zhongguo Zhen Jiu*.

[14] Parajuli N. P., Acharya S. P., Dahal S. (2017). Epidemiology of device-associated infections in an intensive care unit of a teaching hospital in Nepal: a prospective surveillance study from a developing country. *American Journal of Infection Control*.

[15] Esnault P., Nguyen C., Bordes J. (2017). Early-onset ventilator-associated pneumonia in patients with severe traumatic brain injury: incidence, risk factors, and consequences in cerebral oxygenation and outcome. *Neurocritical Care*.

[16] Titsworth W. L., Abram J., Fullerton A. (2013). Prospective quality initiative to maximize dysphagia screening reduces hospital-acquired pneumonia prevalence in patients with stroke. *Stroke*.

[17] Maeshima S., Osawa A., Yamane F., Ishihara S., Tanahashi N. (2014). Association between microbleeds observed on T_2_^∗^-weighted magnetic resonance images and dysphagia in patients with acute supratentorial cerebral hemorrhage. *Journal of Stroke and Cerebrovascular Diseases*.

[18] Jia X. F., Hong Z., Fan J. H., Zhang Y. M. (2016). Clinical effect of mechanical fragmentation combined with recombinant tissue plasminogen activator artery thrombolysis on acute cerebral infarction. *Journal of Biological Regulators and Homeostatic Agents*.

[19] Vial F., Brunser A., Lavados P., Illanes S. (2016). Intraventricular bleeding and hematoma size as predictors of infection development in intracerebral hemorrhage: a prospective cohort study. *Journal of Stroke and Cerebrovascular Diseases*.

[20] Lahiri S., Navi B. B., Mayer S. A. (2015). Hospital readmission rates among mechanically ventilated patients with stroke. *Stroke*.

[21] Zeng Y., Liu J. X., Yan Z. P., Yao X. H., Liu X. H. (2015). Potential microRNA biomarkers for acute ischemic stroke. *International Journal of Molecular Medicine*.

[22] Wang W. M., An X. S., Zhang X. C. (2016). Differential analysis of clinical efficacy on patients with serious infection in ICU by different meropenem regimens. *Pakistan Journal of Pharmaceutical Sciences*.

[23] Tabaeian S. M., Yazdannik A., Abbasi S. (2017). Compliance with the standards for prevention of ventilator-associated pneumonia by nurses in the intensive care units. *Iranian Journal of Nursing and Midwifery Research*.

[24] Valencia A. M., Vallejo C. E., Alvarez A. L., Jaimes F. A. (2017). Attenuation of the physiological response to infection on adults over 65 years old admitted to the emergency room (ER). *Aging Clinical and Experimental Research*.

[25] Rose L., Istanboulian L., Allum L. (2017). Patient- and family-centered performance measures focused on actionable processes of care for persistent and chronic critical illness: protocol for a systematic review. *Systematic Reviews*.

[26] Hao Y., Zhang G., Han B. (2017). Prospective evaluation of respiratory health benefits from reduced exposure to airborne particulate matter. *International Journal of Environmental Health Research*.

[27] Rios-Toro J. J., Marquez-Coello M., Garcia-Alvarez J. M. (2017). Soluble membrane receptors, interleukin 6, procalcitonin and C reactive protein as prognostic markers in patients with severe sepsis and septic shock. *PLoS One*.

